# Metal Organic Framework-Polyethersulfone Composite Membrane for Iodine Capture

**DOI:** 10.3390/polym12102309

**Published:** 2020-10-09

**Authors:** Po-Hsiang Tang, Pamela Berilyn So, Kueir-Rarn Lee, Yu-Lun Lai, Cheng-Shiuan Lee, Chia-Her Lin

**Affiliations:** 1 Department of Chemistry, National Taiwan Normal University, Taipei 11677, Taiwan; fetivear@gmail.com; 2 Department of Chemistry, Chung Yuan Christian University, Taoyuan City 32023, Taiwan; pbtiuso@gmail.com; 3 R&D Center for Membrane Technology, Department of Chemical Engineering, Chung Yuan University, Chung-Li 32023, Taiwan; krlee@cycu.edu.tw; 4 Green Energy and Environment Research Laboratories, Industrial Technology Research Institute, Hsinchu 31040, Taiwan; yulunlai@itri.org.tw (Y.-L.L.); CesiumLi@itri.org.tw (C.-S.L.); 5 R&D Center for Membrane Technology, Chung Yuan Christian University, Taoyuan City 32023, Taiwan

**Keywords:** metal organic framework, iodine capture, mixed matrix membranes, MOF-polymer composite

## Abstract

A variety of metal organic frameworks (MOFs) were synthesized and evaluated for their iodine adsorption capacity. Out of the MOFs tested, ZIF-8 showed the most promising result with an iodine vapor uptake of 876.6 mg/g. ZIF-8 was then incorporated into a polymer, polyethersulfone (PES), at different proportions to prepare mixed matrix membranes (MMMs), which were then used to perform further iodine adsorption experiments. With a mixing ratio of 40 wt % of ZIF-8, the iodine adsorption capacity reached 1387.6 mg/g, wherein an astounding 60% improvement in adsorption was seen with the MMMs prepared compared to the original ZIF-8 powder.

## 1. Introduction

Metal organic frameworks (MOFs) have attracted increasing attention in the industry and academia in recent years due to their remarkable high specific surface area, ordered and adjustable pores [1], as well as their potential for diverse applications including gas adsorption [2], gas storage [3], metal catalysis [4], drug delivery [5], sensors [6], and pollutant capture [7,8]. In addition to this, MOFs have excellent properties such as good thermal stability, good solvent resistance, ease of preparation, large porosity, etc. Its main structure is one-dimensional, two-dimensional, or three-dimensional formed by metal ions or clusters as nodes, connected by organic ligands, and self-assembled in the solution structure.

Iodine is used in a wide range of applications in the agricultural, medical, and industrial fields such as for X-ray contrast media and in liquid-crystal display (LCD) screen production [9]. However, in its vapor phase, it is a highly mobile gas which is toxic and easily spills into the air [10]. Recently, a paper about the quantitative detection of iodine in the stratosphere was reported wherein a link between air quality and ozone loss was established [11]. As the ozone destruction potential of iodine is 600 times more compared to that of chlorine, harmful effects of iodine are detrimental to the environment. In this regard, steps must be taken to prevent the infiltration of iodine into the circulating air. One way to address this problem is through the preparation of multifunctional adsorbent materials which can have high selectivity and adsorption capacity for unwanted pollutants, giving rise to an efficient separation process. MOFs have a great potential for industrial separation due to their high porosity, modular or tunable structures, and inherent chemical properties and selectivity [12,13,14]. These characteristics are highly favored for the preparation of materials for adsorption. 

At present, there have been many literatures on the adsorption of iodine vapor on MOFs. In 2011, the Nenoff group evaluated the capacity of ZIF-8 for the capture and storage of radioactive iodine [15], due to the fact that it is produced during nuclear fuel reprocessing and commonly seen in nuclear reactor accidents. In 2013, the same team also used HKUST-1 to capture iodine vapor [16], which accounted for the highest reported adsorption capacity of a MOF for I_2_ (1.75 g/g).

However, MOF products are mostly polycrystalline and may not be easily applied for industrial use in its bare form. This being said, the preparation of MOFs in a usable form is needed. The traditional method of MOF membrane preparation is done by inorganic thin film deposition. However, high production cost, brittleness, low selectivity, as well as high permeance, make it not the best choice for material preparation [17,18].

Mixed matrix membranes (MMMs) refer to composite membranes prepared by filling inorganic materials as a dispersed phase in a polymer [19]. This type of membrane can combine the excellent properties of organic and inorganic materials, which can perform better than traditional composite membranes. MMMs were first successfully applied in the field of gas separation in the 1980s and then widely used in pervaporation membrane materials [20,21]. With the rapid development of nanoscience, nanomaterials have been increasingly studied from preparation methods to their structure and function control. In the same way, a variety of nanoparticles and biological proteins have been proven to have potential applications in the water treatment process [22,23]. With this, the concept of mixed matrix membranes is not only used as water treatment membranes, but its use has been extended in various fields of application, including gas separation [24]. The design and preparation of MMMs by combining new inorganic nanomaterials and organic materials with traditional polymers has become a research hotspot in the field of membrane separation, and is considered to be the future development direction of high-performance separation membrane materials.

The present study aims to prepare MMMs comprised of polymer-MOF composites. Starting with the synthesis of several MOFs, followed by characterization and evaluation for I_2_ capture, the best candidate was selected to be incorporated into an MMM. The selected MOF was added at different proportions into the polymer matrix for casting then further tested for I_2_ capture in a usable membrane form.

## 2. Materials and Methods

### 2.1. Chemicals

Aluminum chloride hexahydrate (AlCl_3_ 6H_2_O, Alfa (Lancashire, UK), ≥98%), aluminum sulfate octahydrate (Al_2_(SO_4_)_3_ 18H_2_O, J.T. Baker (Radnor, PA, USA), ≥98%), zinc nitrate hexahydrate (Zn(NO_3_)_2_ 6H_2_O, SHOWA (Gyoda, Japan), ≥99%), copper nitrate trihydrate (Cu(NO_3_)_2_ 3H_2_O, SHOWA, ≥99.9%), sodium hydroxide (NaOH, FLUKA-Honeywell (Charlotte, NC, USA), ≥ 99%), zinc oxide (ZnO, Sigma-Aldrich (Saint Louis, MO, USA), ≥99%), 2-methylimidazole (2-MeIM, ACROS ((Geel, Belgium), 99%), 4-benzenedicarboxylic acid (p-H_2_BDC, TCI (Tokyo, Japan), ≥98%), 3-benzenedicarboxylic acid (*m*-H_2_BDC, ACROS, ≥99%), trimesic acid (H_3_BTC, KM3 (New Taipei, Taiwan), ≥98%), fumaric acid (H_2_FUM, Alfa, ≥99%), polyethersulfone (PES, Sigma-Aldrich, average Mw ~35,000), 2-propanol (isopropyl alcohol, IPA, C_3_H_7_OH, J.T. Baker, tech. grade, 99%), methanol (MeOH, CH_3_OH, Merck (Darmstadt, Germany), ≥99.5%), *N*,*N*-dimethylformamide (DMF, C_3_H_7_NO, Merck ≥99.5%), ammonia (NH_4_OH, Merck, 25%), 1-methyl-2-pyrrolidone (NMP, Sigma-Aldrich, ≥99%), ethanol (EtOH, TCI, 99.5%)

### 2.2. Material Synthesis

#### 2.2.1. Synthesis of ZIF-8

ZIF-8 was prepared following a previously published method [25,26] with minor modifications. In brief, 0.5872 g (1.974 mmol) of zinc nitrate hexahydrate (Zn(NO_3_)_2_ 6H_2_O) was dissolved in 12 mL of MeOH while 1.2978 g (15.808 mmol) of the ligand 2-methylimidazole was dissolved in 28 mL of NH_4_OH. The two solutions were mixed and stirred at room temperature for 1 h to obtain the as-synthesized product. This was then washed with 10 mL of MeOH three times, centrifuged, then dried in an oven at 75 °C for 1 day to obtain a white powder.

#### 2.2.2. Synthesis of HKUST-1

HKUST-1 was prepared following the previously published paper [27] with minor modifications. In brief, 41.4894 g (170 mmol) of copper nitrate trihydrate (Cu(NO_3_)_2_ 3H_2_O), 20.0213 g (95 mmol) of H_3_BTC, and 7.0012 g (85 mmol) of zinc oxide (ZnO) were placed into a 2 L glass beaker followed by the addition of 381.5 mL each of EtOH, H_2_O, and DMF (*w:w:w* = 1: 1: 1). The mixture was stirred at room temperature for 6 h to obtain a solid product. This was then washed three times with 100 mL of EtOH and dried in an oven at 120 °C for 1 day to obtain a light blue powder.

#### 2.2.3. Synthesis of MIL-68 (Al)

MIL-68(Al) was prepared following a previously published method [28] with minor modifications. In brief, 0.4829 g (2 mmol) of aluminum chloride hexahydrate (AlCl_3_ 6H_2_O) and 0.1667 g (1 mmol) of ligand terephthalic acid (*p*-H_2_BDC) were placed in a round bottom flask, followed by the addition of 8 mL of IPA. The flask was shaken for 10 min to make it evenly mixed and then refluxed at 100 °C for 1 day to obtain a solid product. This was then washed three times with 10 mL of MeOH and dried in an oven at 75 °C for one day to obtain a white powder.

#### 2.2.4. Synthesis of A520

A520 was prepared following the previously published paper [29] with minor modifications. In brief, 0.7 g (1.05 mmol) of aluminum sulfate octahydrate (Al_2_(SO_4_)_3_ 18H_2_O), 2.447 g (2.11 mmol) of fumaric acid (H_2_FUM), and 2.532 g (6.33 mmol) of sodium hydroxide (NaOH) were placed in a round bottom flask, followed by the addition of 6.6 mL of H_2_O, which was then shaken for 10 min for a uniform mixture. It was then refluxed at 60 °C for 2 h to obtain a solid product. This was then washed three times with 10 mL of H_2_O and dried in an oven at 120 °C for 1 day to obtain a white powder.

#### 2.2.5. Synthesis of CAU-10

CAU-10 was prepared following the previously published paper [30] with minor modifications. In brief, 0.505 g (0.75 mmol) of aluminum sulfate octahydrate (Al_2_(SO_4_)_3_ 18H_2_O), 0.132 g (0.79 mmol) of isophthalic acid (*m*-H_2_BDC), 2.5 mL of H_2_O, and 0.625 mL of DMF were mixed in a round bottom flask and were shaken for 10 min to get a uniform mixture. This was refluxed at 100 °C for 2 days to obtain a solid product. This was then washed three times with 10 mL of DMF, then washed three times with 10 mL of H_2_O, and dried in an oven at 120 °C for 1 day to obtain a white powder.

Due to the porous nature of the synthesized MOFs materials, guest molecules (from unreacted metal sources, organic ligands, or the solvent used for synthesis) often stay in the pores of the frameworks. Through appropriate purification and activation steps, guest molecules in the pores can be effectively removed and the porous properties of the MOFs synthesized can be improved. In this experiment, the solvent exchange was used for the purification process, which is done by washing the newly synthesized MOF with solutions that can be easily removed by heating. This is to be able to completely replace the solvent trapped in the pores of the framework and then efficiently remove the new solvent by activation at 120 °C under a vacuum for 1 day.

### 2.3. Characterization of Synthesized MOFs

Characterization of the synthesized MOFs was done using a Bruker D8 advance powder x-ray diffractometer (PXRD) with Cu Kα radiation (λ = 1.54056 Å) at room temperature scanning at a 2θ range from 2° to 40°. BET surface areas of the synthesized MOFs were computed by measuring the nitrogen adsorption using the Micromeritics ASAP2020 surface area and porosimetry system. Prior to analysis, the MOFs were degassed at 120 °C for 24 h.

### 2.4. Preparation of Membrane Film

The polymer polyethersulfone (13.33 g) was dissolved in 80 g (77.69 mL) of 1-methyl-2-pyrrolidone (NMP) in a 100 mL screw top glass bottle then stirred in an oil bath at 60 °C for 1 day. This was followed by the addition of 6.67 g of ZIF-8 and was stirred for a uniform dispersion. When a uniform dispersion was obtained, the bottle was removed from the oil bath and cooled down to room temperature.

#### 2.4.1. Preparation of Wet-Film (ZIF-8@PES w)

The casting solution was poured on an A4 size glass plate and the film was leveled using a casting rod with a 500 μm gap. After casting, the membrane film was directly immersed in a water coagulation bath. After film formation, the MMM can be directly peeled off from the glass plate to form a single separated film. After separation, the film was immersed in water to replace the NMP in the film. The water was replaced once a day for a total of 3 days to facilitate the solvent exchange. After drying at 45 °C for 1 day, a white MMM was obtained which is denoted as ZIF-8@PES w.

#### 2.4.2. Preparation of Dry-Film (ZIF-8@PES d)

The resulting casting solution is poured on an A4 size glass plate and the film was leveled using a casting rod with a 500 μm gap. After casting, the glass plate is directly dried in an oven at 60 °C for 2 h. After solvent evaporation, the white MMM can be peeled off directly from the glass plate which is denoted as ZIF-8@PES d.

### 2.5. Preparation of MMM with Different Mixing Ratios

In the preparation of the membrane casting solution, the dry-free concept is used so the synthesized MOF was not dried prior to incorporation. However, as it is necessary to know the ratio of the incorporated MOF to the amount of polymer used, the yield for ZIF-8 synthesis was computed (77.9% based on metal). Using this data, the amount of starting materials for the synthesis of ZIF-8 needed for the casting solution was computed. The synthesized ZIF-8 was washed with NMP three times to replace the guest molecules in the pores with NMP, preventing a “two-solvent system”, which can cause pre-mature film formation prior to membrane casting. Different mixing ratios were prepared as shown in Table 1.

The preparation of the casting solution was done by first dissolving the high molecular weight PES in 20 g (19.42 mL) of NMP in a 100 mL screw cap glass bottle stirring it in an oil bath at 60 °C for 1 day. After dissolution, the remaining NMP was used to transfer the undried ZIF-8 into the glass bottle and stirred to get a uniform dispersion.

### 2.6. Characterization of MMM

#### 2.6.1. Powder X-ray Diffraction (PXRD)

The prepared MMM, ZIF-8@PES d, and ZIF-8@PES w, were cut into 0.5 cm × 0.5 cm sized squares and affixed to an acrylic stage using clay, then they were characterized using the powder X-ray diffractometer for preliminary structure identification.

#### 2.6.2. Thermogravimetric Analysis (TGA)

ZIF-8@PES was cut into small pieces and 10 mg was placed in a platinum pan for testing. TGA was done under nitrogen atmosphere with a flow rate of 60 mL/min. Heating from 30 to 800 °C with a heating rate of 10 °C/min, the percent weight loss was recorded.

#### 2.6.3. Scanning Electron Microscopy with Energy Dispersive X-ray Analysis (SEM-EDX)

Scanning electron microscopy (SEM) images were captured on a JEOL JSM-7600F FE-SEM (Tokyo, Japan) and energy-dispersive x-ray analysis (EDX) were done with an Oxford Instruments X-max^80^ EDS.

### 2.7. Iodine Capture

As I_2_ gas is strongly oxidizing, glass containers were used for the experiment. Different MOFs were placed into a 10 mL sample bottle, and then inside a 1 L wide-mouth beaker with cover. Iodine was then added into the beaker, closed, and then placed on a hot plate to sublime the iodine and expose the MOFs to the I_2_ vapors.

For the MMMs, ZIF-8@PES w and ZIF-8@PES d film were separately placed in a 5 cm glass petri dish, which is placed inside another 10 cm glass petri dish. Iodine was then placed on the 10 cm glass petri dish, covered, then heated on a hot plate to sublime the iodine and expose the membrane to I_2_ vapors with an exposure time of up to 8 h. The membranes were weighed at each time point and the weight difference of the film were recorded. Using the gravimetric method, the adsorption capacity was calculated to obtain the adsorption curve.

## 3. Results and Discussion

**Figure 1:**
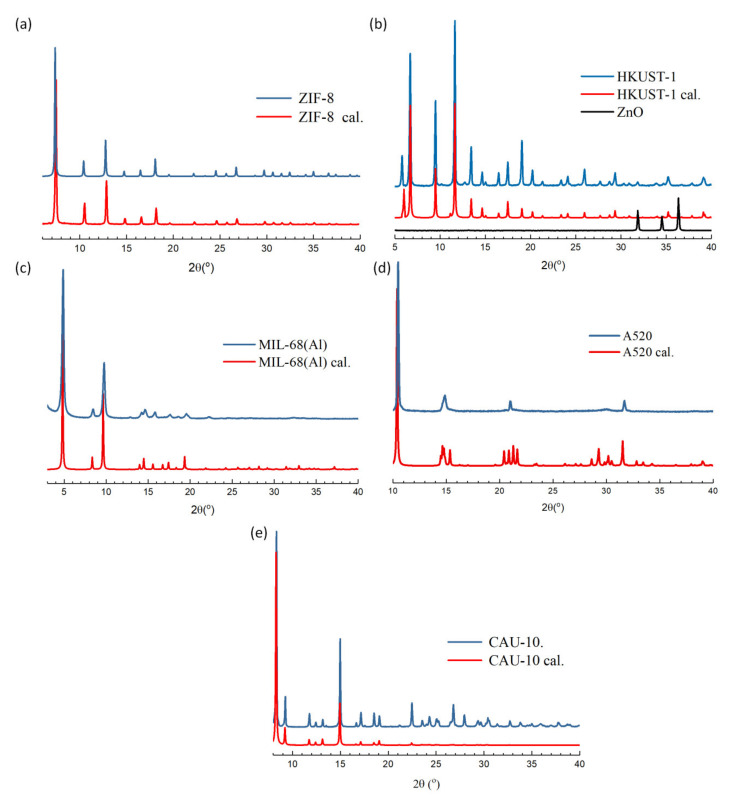
Powder pattern of (**a**) ZIF-8, (**b**) HKUST-1, (**c**) MIL-68(Al), (**d**) A520, and (**e**) CAU-10.

Characterization of the synthesized MOFs were done using a powder diffractometer and the measured PXRD patterns were compared with theoretical data as shown in Figure 1. All synthesized MOFs are in good agreement with the calculated values. In addition, the characteristic peaks of ZnO as a template for crystal growth in HKUST-1 synthesis are almost absent, indicating that the reaction is complete and the starting material is not present in the product. Nitrogen adsorption desorption isotherms of the synthesized MOFs are presented in Figure S1 with their computed BET surface area as well as their pore size distribution (Table S1) are comparable with the published literature.

**Figure 2:**
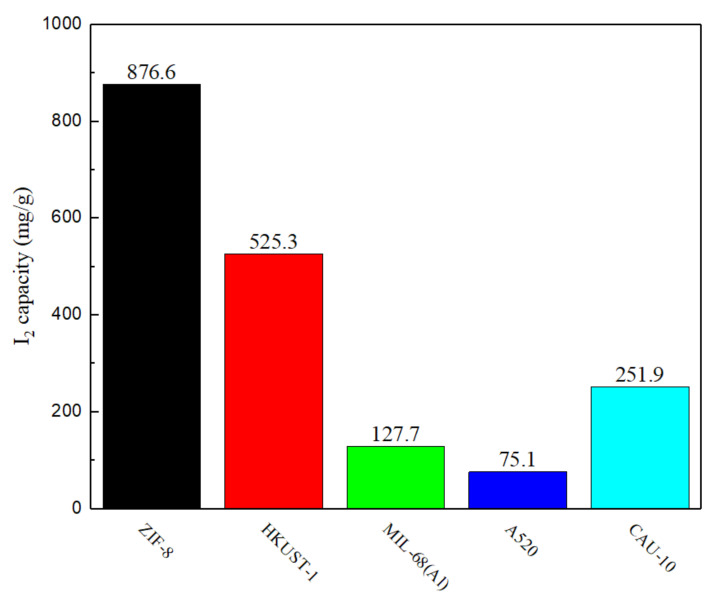
MOF captures I_2_ adsorption results.

ZIF-8 has the highest iodine adsorption capacity, reaching 876.6 mg/g, followed by HKUST-1 with 525.3 mg/g, CAU-10, MIL-68, then A520, with 251.9, 127.7, and 75.1 mg/g, respectively (Figure 2). ZIF-8 exhibited an outstanding ability to capture iodine vapor compared to the other four types of MOF because from the perspective of its structure, it can be seen that ZIF-8 possesses a three-dimensional cage-like structure. When I_2_ molecules are adsorbed into the pores, this type of structure makes it difficult to leave. Contrarily, HKUST-1, MIL-68(Al), A520, and CAU-10 have two-dimensional pore and tunnel structures, thereby making molecules easier to leave through channels in the frameworks. This makes it difficult to trap iodine molecules, resulting in poor adsorption and capture ability. After the I_2_ capture, the sample changes from white or blue color to brown, as shown in the supporting information (Figure S2).

**Figure 3:**
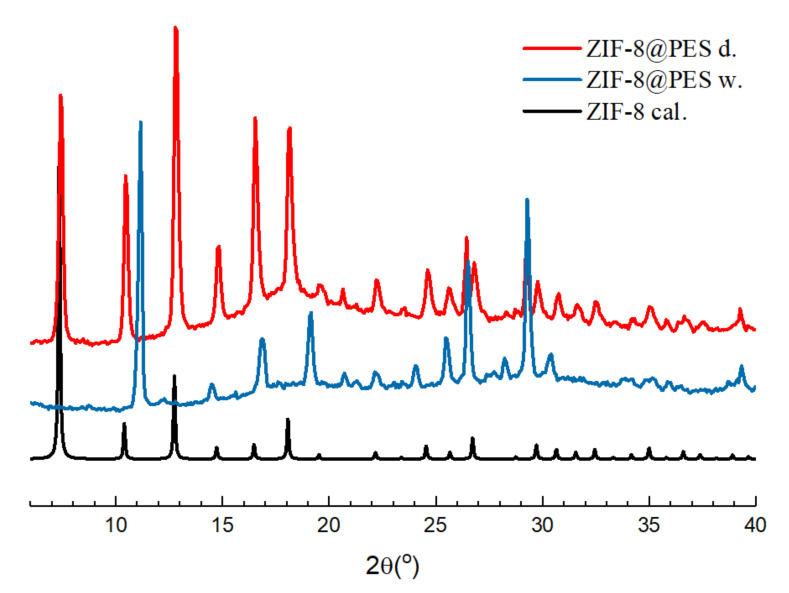
Powder pattern of ZIF-8 membrane prepared using different methods.

As ZIF-8 showed the highest I_2_ adsorption capacity, it was selected to be incorporated into a membrane. The prepared MMM, ZIF-8@PES d, and ZIF-8@PES w, were cut into 0.5 cm × 0.5 cm sized squares and affixed to the acrylic stage using clay. Then, they were characterized using the powder X-ray diffractometer for preliminary structure identification (Figure 3) and were compared with the theoretical diffraction spectrum of ZIF-8.

It can be observed that the diffraction spectrum of ZIF-8@PES d is comparable with the theoretical diffraction spectrum of ZIF-8 while the diffraction spectrum of ZIF-8@PES w is distinctly different with the calculated data. It is speculated here that although ZIF-8 has a certain degree of water stability, soaking ZIF-8 for too long will make the structure collapse. As water stability is usually evaluated by PXRD after soaking MOFs for a certain period of time [31], 0.1 g of ZIF-8 was immersed in 10 mL of H_2_O in a 20 mL sample bottle and stirred for 1 day at room temperature. It was then lyophilized for 1 day to obtain a white powder, ZIF-8 w.

**Figure 4:**
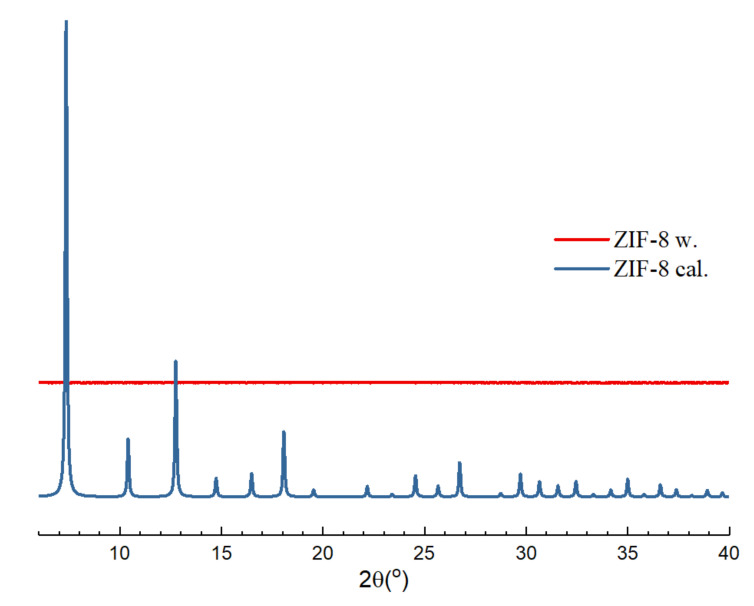
Powder x-ray diffractometer (PXRD) pattern of ZIF-8 after water immersion (ZIF-8 w) in comparison with the as synthesized ZIF-8.

The PXRD pattern of the dried ZIF-8 w powder was then measured with a scanning angle of 6 to 40° (Figure 4). It can be observed from Figure 4 that after stirring in H_2_O for 1 day, the structure of ZIF-8 collapsed exhibiting no signs of crystallinity. Similarly, the same is observed with that of ZIF-8@PES w where there is no characteristic peak of ZIF-8, due to the immersion of the membrane in H_2_O for solvent exchange, which caused the ZIF-8 structure to collapse.

**Figure 5:**
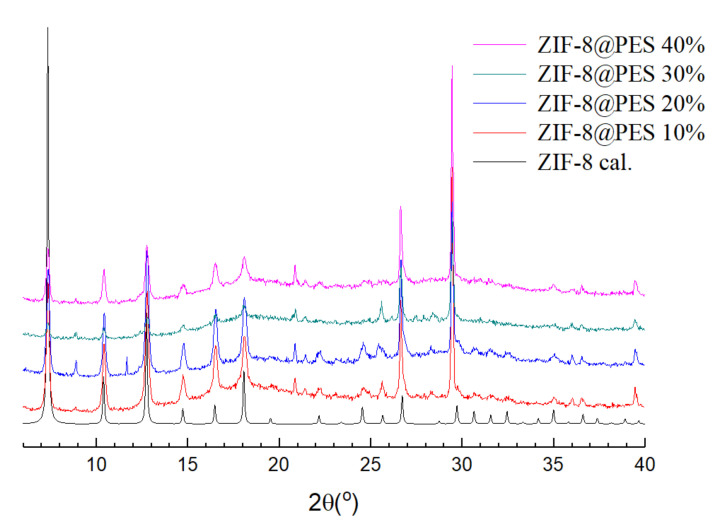
PXRD pattern of prepared mixed matrix membranes (MMMs) at different mixing ratios of ZIF-8.

The MMMs prepared using different mixing ratios were also measured for their PXRD patterns and compared with the theoretical diffraction spectrum of ZIF-8 (Figure 5). It can be observed that PXRD patterns of the prepared MMMs are in good agreement with the calculated data with the exception of the appearance of a new peak at 28.5°, which is from PES as seen in the PXRD pattern of plain PES membrane in Figure S3.

**Figure 6:**
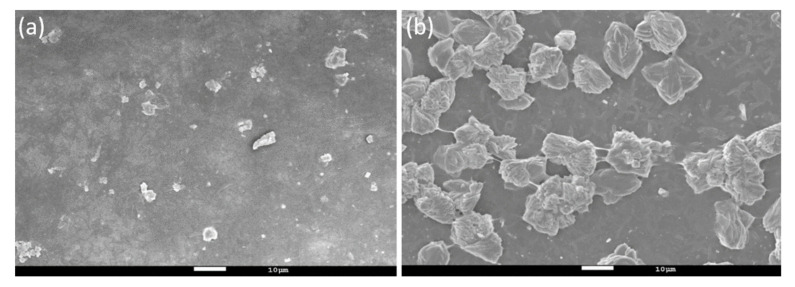
SEM image of (**a**) ZIF-8@PES w and (**b**) ZIF-8@PES d.

The surface morphology of the MMM prepared using the wet and dry method were compared using SEM at a magnification of 1 × 10^3^ times (Figure 6). It can be seen in Figure 6 that there are many granular materials in ZIF-8@PES w with a particle size falling between 1–5 μm. The same is true for ZIF-8@PES d but with particle sizes ranging between 10–20 μm. With this, it can be observed that the MMM made by the dry method causes an obvious agglomeration phenomenon (larger particles). In ZIF-8@PES w, the film is formed directly by immersing in water so the relative position between the polymer and the MOF is fixed, which would make it less prone to agglomeration, but in ZIF-8@PES d, the slow evaporation of NMP may cause uneven dispersion of MOF in the casting fluid, which makes it easier to agglomerate.

**Figure 7:**
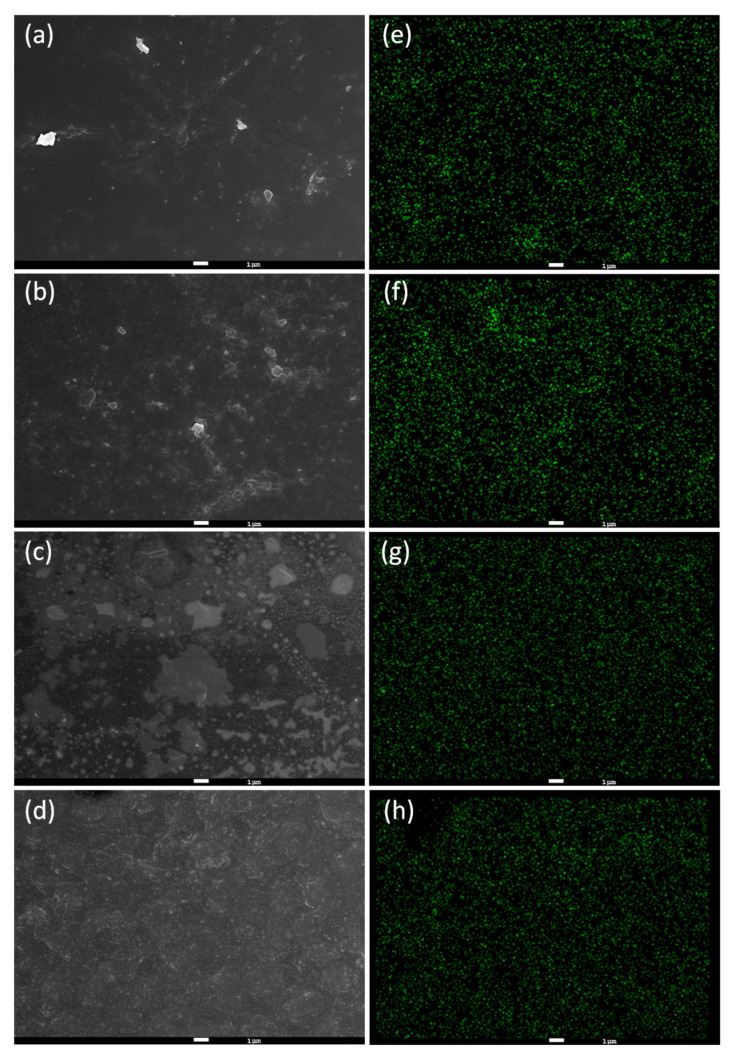
Surface morphology of ZIF-8@PES prepared using different mixing ratios at high magnification. (**a**–**d**) SEM micrograph of 10, 20, 30, and 40 wt %, respectively, and (**e**–**h**) SEM-EDX of 10, 20, 30, and 40 wt %, respectively (Green: Zinc).

Observation of the surface morphology of the MMM was done using SEM at a magnification of 5 × 10^3^ (Figure 7a–d). Granular particles with sizes around 1 μm can be observed on the surface of the ZIF-8@PES and the number of particles increases with the increase of the MOF mixing ratio. In order to prove that the dry-free method can uniformly disperse the MOF in the membrane, the films were subjected to SEM-EDX characterization. In Figure 7e–h, the green dots represent the zinc metal from ZIF-8 and it can be seen they are evenly distributed on the film. With this, it can be inferred that the dry-free method of MOF incorporation was able to uniformly disperse the MOF in the casting solution.

**Figure 8:**
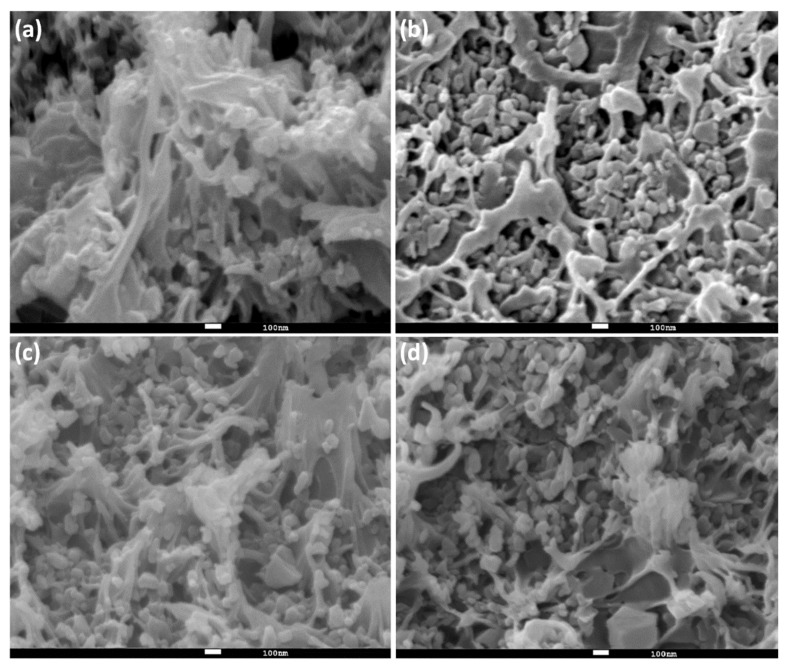
Cross-section SEM of ZIF-8@PES with different MOF mixing ratios (**a**) 10%, (**b**) 20%, (**c**) 30%, and (**d**) 40%.

Under a magnification of 5 × 10^4^ (Figure 8), it can be observed that ZIF-8@PES is comprised of a combination of materials where the polymer resembles a fibrous or thread-like shape, while the ZIF-8 is granular. Even at a high magnification, it can be observed that the granular material is uniformly dispersed in the MMM.

**Figure 9:**
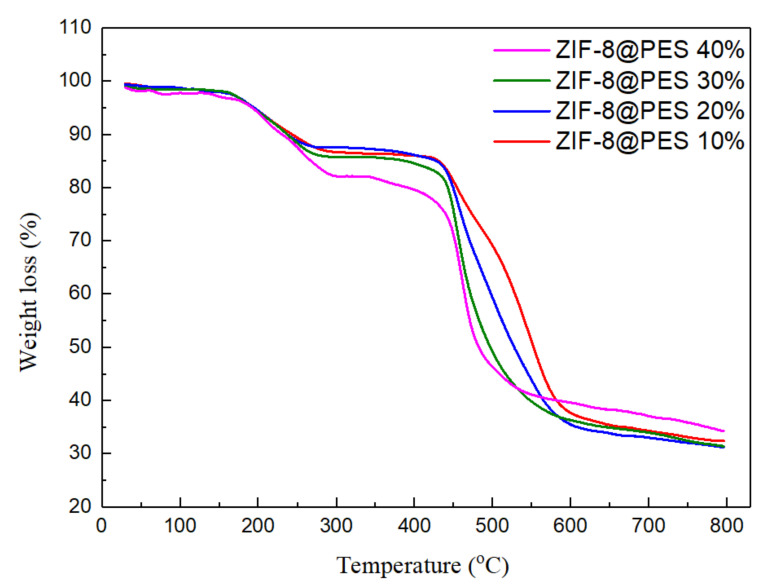
TGA curve of prepared MMMs at different mixing ratios of ZIF-8.

As shown in Figure 9, it can be observed that ZIF-8@PES starts to thermally decompose at 200 °C. This is attributed to the weight loss caused by the departure of NMP from the film. In the same way, the weight loss at 450 °C and after 600 °C is attributed to the decomposition of ZIF-8 and PES, respectively, as seen in Figure S4 showing the TGA of ZIF-8 and PES alone. The remaining 30 wt % of material is presumably the metal oxide of zinc (ZnO).

**Figure 10:**
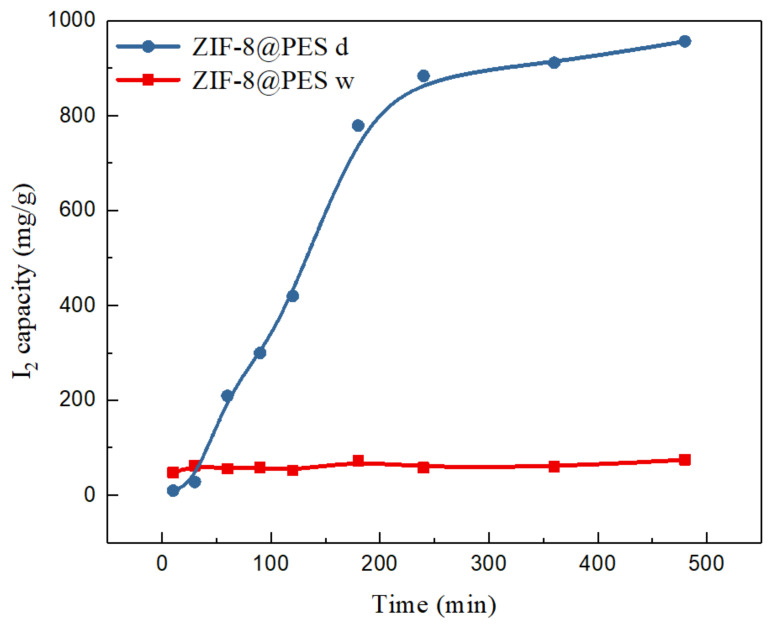
I_2_ adsorption curve of the prepared MMMs (ZIF-8@PES d in blue and ZIF-8@PES w in red).

The data for iodine capture of the prepared MMMs are shown in Figure 10. It can be observed from the adsorption curve that ZIF-8@PES w reaches the adsorption saturation within 10 min, while for ZIF-8@PES d, the adsorption saturation was only reached after 4 h. Comparing the two MMMs prepared, ZIF-8@PES d has a better ability to capture I_2_ vapors. The iodine adsorption capacity of ZIF-8@PES d is 956.89 mg/g, while for ZIF-8@PES w, it is only 74.53 mg/g. The reason for the huge difference in adsorption capacity is due to preservation of the ZIF-8 structure in the membranes. As we see in the PXRD patterns, the distinctive peaks for ZIF-8 can barely be seen in the ZIF-8@PES w as compared with ZIF-8@PES d where it shows the full diffraction spectrum of the calculated ZIF-8. The inorganic material ZIF-8 with a high specific surface area is mainly responsible for the I_2_ capture. However, during the preparation of the wet film, the structure of ZIF-8 collapsed, which resulted in the decrease of the iodine adsorption capacity. Images of the prepared MMMs before and after I_2_ adsorption are seen in the supporting information where a distinct color change can be observed (Figure S5). To rule out any discrepancies, PES polymer membranes were also made without ZIF-8 by the dry method and evaluated for its I_2_ adsorption capacity (468.1 mg/g), as shown in Figure S6.

**Figure 11:**
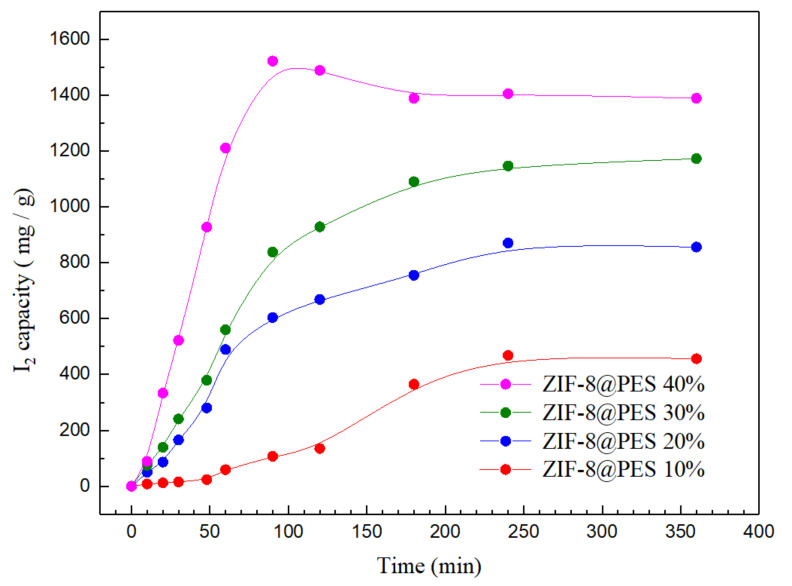
I_2_ adsorption curve of prepared MMMs at different mixing ratios of ZIF-8.

To further prove the significance of the ZIF-8 component for I_2_ capture, MMMs with different weight ratios of ZIF-8 were prepared using the dry method and tested for their I_2_ adsorption capacity, which are shown in Figure 11. It can be observed from the adsorption curve that ZIF-8@PES with different mixing ratios reaches the saturation point at around 4 h. Among the prepared MMMs, ZIF-8@PES 40 wt % has exhibited supersaturation during the initial part of the adsorption process at about 80 min. As the mixing ratio of MOF increases, the adsorption amount of I_2_ also increases gradually. It is worth noting that when the 30 and 40 wt % of MOF was incorporated into the MMM, the I_2_ capture increased to 1171.9 and 1387.6 mg/g, respectively, exhibiting an adsorption capacity that is far better than that of ZIF-8 itself, showing a higher I_2_ adsorption capacity compared to other MOF-polymer composites (Table S2). This astounding increase in the I_2_ capture is attributed to two possible reasons: (1) Upon observation of the surface morphology of ZIF-8@PES, interfacial gaps can be seen between ZIF-8 and PES leading to a void volume leading to an increase in the I_2_ adsorption capacity; and (2) as the dry-free method is used for the MOF incorporation into the membrane, ZIF-8 becomes uniformly dispersed in the matrix also leading to an increase in the I_2_ adsorption capacity.

Moreover, compared with the initial MMM preparation where 33% of dried ZIF-8 was used for incorporation, the capacity for I_2_ adsorption was only 956.89 mg/g. This is significantly lower than the MMM prepared using the dry-free method with only 30 wt % of ZIF-8 but exhibits a higher I_2_ adsorption (1171.9 mg/g). With this, it can be inferred that the uniform dispersion of ZIF-8 on the membrane by the dry-free incorporation is effective in increasing the I_2_ adsorption capacity.

## 4. Conclusions

The I_2_ adsorption capacity of various MOFs were determined, wherein ZIF-8 gave the highest adsorption capacity of up to 876.6 mg/g. With this, it was selected as the MOF to be incorporated into the MMM to be prepared. Two different methods were utilized for membrane preparation: Wet and dry method. As the wet method causes the ZIF-8 structure to collapse, the dry method was chosen. Using SEM-EDX, it was confirmed that the dry-free MOF incorporation method can uniformly disperse ZIF-8 in the MMM without causing agglomeration, thus, it was utilized giving an efficient uniform dispersion of ZIF-8 in the membrane. Different MOF mixing ratios were prepared to evaluate the effect of increasing the amount of ZIF-8 in the MMMs where it was proven that a higher amount of ZIF-8, up to 40 wt %, gave a better I_2_ adsorption capacity. The result of the thermogravimetric analysis shows that the overall thermal stability of the film is about 200 °C. In addition, the blending of ZIF-8 and PES yields an interfacial space increasing the void volume of the MMMs, thereby increasing the ability of the MMMs to adsorb the I_2_ gas, giving a better adsorption capacity (1387.6 mg/g) than that of the ordinary ZIF-8 powder (876.6 mg/g) and PES alone (468.1 mg/g); an astounding improvement of the adsorption capacity by nearly 60%.

## Figures and Tables

**Table 1:** Metal organic framework (MOF) ratios for preparation of casting solution.

MOF Ratio	ZIF-8	PES	NMP	Membranes
10%	1 g	9 g	40 g	ZIF-8@PES 10%wt
20%	2 g	8 g		ZIF-8@PES 20%wt
30%	3 g	7 g		ZIF-8@PES 30%wt
40%	4 g	6 g		ZIF-8@PES 40%wt

## Supplementary Materials

The following are available online at https://www.mdpi.com/2073-4360/12/10/2309/s1. Figure S1: N_2_ adsorption isotherms and differential pore volume graphs of synthesized MOFs; Table S1: BET surface area and differential pore distribution of synthesized MOFs; Figure S2: Images of the synthesized MOFs before and after I_2_ capture; Figure S3: PXRD pattern of plain PES membrane prepared using the dry method; Figure S4: TGA curve of ZIF-8 and PES d; Figure S5: Images of the prepared MMMs before and after I_2_ capture; Figure S6: I_2_ adsorption curve of the PES membrane prepared using the dry method; Table S2: Comparison of I_2_ adsorption capacities of MOF-polymer composites.
